# Social Differentiation in Common Bottlenose Dolphins (*Tursiops truncatus*) that Engage in Human-Related Foraging Behaviors

**DOI:** 10.1371/journal.pone.0170151

**Published:** 2017-02-01

**Authors:** Carolyn J. Kovacs, Robin M. Perrtree, Tara M. Cox

**Affiliations:** Marine Sciences Program, Department of Marine & Environmental Sciences, Savannah State University, Savannah, Georgia, United States of America; University of Missouri Columbia, UNITED STATES

## Abstract

Both natural and human-related foraging strategies by the common bottlenose dolphin (*Tursiops truncatus*) have resulted in social segregation in several areas of the world. Bottlenose dolphins near Savannah, Georgia beg at an unprecedented rate and also forage behind commercial shrimp trawlers, providing an opportunity to study the social ramifications of two human-related foraging behaviors within the same group of animals. Dolphins were photo-identified via surveys conducted throughout estuarine waterways around Savannah in the summers of 2009–2011. Mean half-weight indices (HWI) were calculated for each foraging class, and community division by modularity was used to cluster animals based on association indices. Pairs of trawler dolphins had a higher mean HWI (0.20 ± 0.07) than pairs of non-trawler dolphins (0.04 ± 0.02) or mixed pairs (0.02 ± 0.02). In contrast, pairs of beggars, non-beggars, and mixed pairs all had similar means, with HWI between 0.05–0.07. Community division by modularity produced a useful division (0.307) with 6 clusters. Clusters were predominately divided according to trawler status; however, beggars and non-beggars were mixed throughout clusters. Both the mean HWI and social clusters revealed that the social structure of common bottlenose dolphins near Savannah, Georgia was differentiated based on trawler status but not beg status. This finding may indicate that foraging in association with trawlers is a socially learned behavior, while the mechanisms for the propagation of begging are less clear. This study highlights the importance of taking into account the social parameters of a foraging behavior, such as how group size or competition for resources may affect how the behavior spreads. The positive or negative ramifications of homophily may influence whether the behaviors are exhibited by individuals within the same social clusters and should be considered in future studies examining social relationships and foraging behaviors.

## Introduction

Optimal foraging theory indicates that organisms should maximize their caloric intake while minimizing time spent searching for and obtaining each food item [1]. Intra-specific competition can reduce the value of an otherwise optimal food source by depleting the abundance of a specific prey type, thus increasing search time. Therefore, specialists within a population may target prey that are less abundant or provide less energy if the predator can maximize intake rate by reducing competition. Differentiation of foraging strategies can reduce competition between individuals by reducing the number of predators targeting each prey source [2].

In some cases foraging specialization and resource partitioning can lead to social segregation. Killer whales (*Orcinus orca*) in the eastern North Pacific are assumed to have all belonged to a single form, and then over time specialized into two reproductively isolated forms currently known as transients, which feed on mammals, and residents, which feed on fish [3]. Therefore, individual variation in niche use can affect rates of interaction between individuals that result in population level effects [4]. Common bottlenose dolphins (*Tursiops truncatus*) in Florida Bay foraged by either deep diving or mud ring feeding, and only 3.7% of all sightings had both ecotypes present [5]. The limited encounter rate decreased reproductive opportunities, which could cause subpopulation divergence [5].

One type of foraging specialization by cetaceans that has been observed in many areas worldwide is the use of food sources that are provided directly or indirectly by humans. This human-related foraging can lead to social segregation, as observed between bottlenose dolphins (*Tursiops spp*.) that foraged in association with trawlers and those that did not in Moreton Bay, Australia [6] and in North Carolina, USA [7]. In many cases the social groups are not completely segregated, but there is social clustering of groups based on foraging behaviors. Common bottlenose dolphins have shown higher rates of association with other individuals engaged in the same foraging class when part of the population was known to engage in foraging around anthropogenic feeding stations such as trawlers or aquaculture farms [8–9] or cooperative fishing with fishermen [10].

Learning can occur on one’s own via trial and error (asocial learning), or by learning from conspecifics (social learning). The learning of many foraging behaviors may not necessitate innovation or social transmission, but simply individuals interacting with their environments, as many behaviors have been seen in multiple parts of the world [11–12]. However, the appearance of new behaviors that have spread throughout populations point to social learning as a primary method of dispersal [13–15]. Social learning has been documented in many animals, including bats [16], primates [17–18], and cetaceans [14–15]. If individual or asocial learning is occurring, then it may be more common to have mixed groups, comprised of individuals that engage in different foraging strategies [19]. This may increase overall feeding efficiency of all members, as they will not be competing with one another. Conversely, if social learning were responsible for a behavior, one would expect to see groups of individuals that spend the most time together demonstrating similar behaviors.

Dolphin-human interaction behaviors, including begging, depredating, patrolling, provisioning, and scavenging as defined previously [20–22], are common in the waterways near Savannah, Georgia. Common bottlenose dolphins exhibited human-interaction behaviors on 69.6% of days and 23.5% of sightings near Savannah in 2009 and 2010 [22]. The most common human-interaction behavior observed was begging (22.4% of sightings) [22]. In addition, 59 individuals were observed interacting with humans at least once, yielding 20.1% of identified animals in the population known to interact with humans [22].

Although begging has been observed in other studies of bottlenose dolphins in the southeastern United States as well as Australia, the rates observed in Savannah are considerably higher than any others that have been reported [21–23]. Savannah also has a well-established shrimp trawl fishery that common bottlenose dolphins have associated with since at least the early 1970s. Dolphins near Savannah follow behind active trawlers, feeding on fish that are caught in or stirred up by the nets. In addition, many of the dolphins feed on the bycatch that is discarded off the trawler [24]. Similar trawler-associated foraging has been observed worldwide [6,25–28].

The presence of both begging and associating with shrimp trawlers within the same study area provides a unique opportunity to examine the social structure of a group of dolphins as it pertains to two different human-related foraging behaviors. This study attempts to determine whether the social structure of common bottlenose dolphins near Savannah, Georgia is differentiated based on begging or associating with shrimp trawlers and the implications of any such differentiations.

## Methods

### Surveys

**Fig 1:**
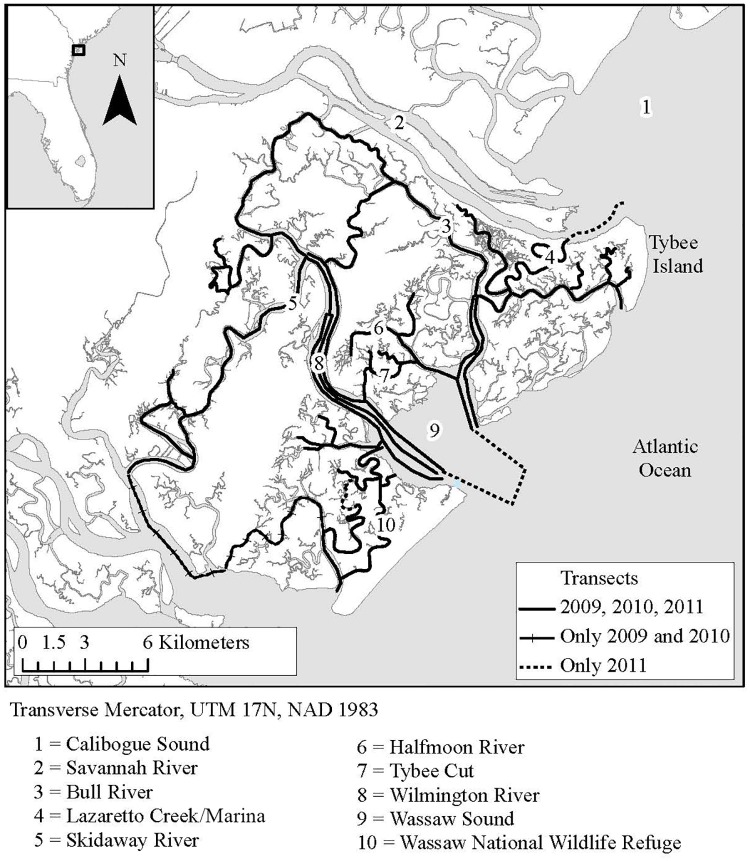
The study area, located near Savannah, Georgia. Transects surveyed are denoted for each year of surveys from 2009–2011.

Photo-identification surveys were conducted in the inshore waters near Savannah, Georgia from April through August 2009 and May through August 2010 and 2011. Surveys were conducted from a 6.7 m Boston Whaler at speeds of 33–41 km/h. While on effort, a minimum of two observers scanned the water looking for dolphins. A total of 635 group sightings were made on 99 survey days. The study area contained sounds, rivers, and creeks surrounded by salt marshes and covered approximately 340 km^2^ centered at 80.00°W, 32.01°N (Fig 1). In addition, surveys up to 3 km offshore were conducted in 2011 (Fig 1). Photographs and data of protected common bottlenose dolphins (*Tursiops truncatus*) were collected in accordance with the Marine Mammal Protection Act under National Marine Fisheries Service Letter of Confirmation number 14219 issued to T. Cox, and all areas accessed were public waterways.

When dolphins were sighted, the time and position were recorded using a Garmin^®^ GPS Map76, and the boat was moved parallel and at a similar speed to the dolphins. Photographs were taken of the dorsal fin of each individual in the group using digital SLR cameras (Nikon^®^ D90 and Canon^®^ EOS 40D and 60D) with 70–300 mm or 70–400 mm zoom lenses. All animals within 100 m of one another and moving in the same direction or engaging in the same behavior were included within the same sighting [29]. The number of individuals and number of calves (less than 2/3 the length of adults, surfacing with an adult) in the sighting, and behaviors observed were recorded [29]. All observations of individuals begging were recorded and photos of the dorsal fin were taken for identification purposes when possible [30]. Begging was defined as a dolphin surfacing parallel to the boat with the ventral side towards the vessel within 2 m of the boat, or surfacing with its head out of the water oriented towards the vessel within 10 m of the vessel [22].

Targeted surveys of dolphins associating with shrimp trawlers occurred whenever an actively trawling vessel was observed in the area. Trawler sightings occurred along inshore survey transects as well as up to 4 km past these transects, outside of Wassaw Sound in 2010 (Fig 1). In 2011, transects were added along this ocean route and into southern Calibogue Sound. Shrimp trawlers are not permitted to trawl within inland waters in Georgia and South Carolina. The majority of sightings around actively trawling vessels occurred at the mouth of Wassaw Sound (n = 11), with 2 additional sightings in southern Calibogue Sound.

Photos were taken of all dolphins that associated with a trawler. For this study, dolphins that followed actively trawling vessels within 150 meters of the stern while the nets were in the water were considered to be dolphins that associated with shrimp trawlers. The distance of 150 m was based on the approximate distance from the stern of the trawler to the end of the net. Only sightings in which all dolphins associated with the trawler were used in analyses to ensure that no dolphins that were within the sighting but engaged in a different behavior, such as socializing, were misidentified as “trawler dolphins.” This distinction only removed one sighting with a large group size from the study.

### Photo-ID and analysis

The best images of each individual were graded for fin distinctiveness and photographic quality, according to the protocols developed for the Mid-Atlantic Bottlenose Dolphin Photo-Identification Catalog [30]. Distinctive animals with one excellent image or two good images from different days were added to a photo-identification catalog for the Savannah area.

A discovery curve determined that dolphins that begged displayed the behavior within the first 6 sightings on separate days (S1 Fig) [31]. Therefore, dolphins in the catalog that were seen on ≥ 6 field days were categorized based on observed human interactions. If a dolphin was seen associating with a trawler with nets in the water it was considered a trawler dolphin. Dolphins seen begging at any vessel (including recreational boats, the research boat, and commercial crab or shrimp boats) or a dock were labeled as beggars. Confirmed non-beggars and confirmed non-trawlers were individuals sighted on ≥ 6 field days without having ever been seen begging or associating with a trawler, respectively. Some animals seen on ≥ 6 field days were considered “unknown” for beg status, because even though they were sighted on ≥ 6 field days they could not be confirmed as not begging (i.e. they were seen in large groups with begging observed but the beggars could not be identified). Beg status and trawler status were assigned separately for each individual; therefore, each individual could be classified as a) beggar, non-trawler; b) non-beggar, trawler; c) beggar and trawler; d) non-beggar, non-trawler; e) unknown beg status, trawler; or f) unknown beg status, non-trawler. The percentage of dolphins in the catalog seen on ≥ 6 field days that begged, associated with trawlers, engaged in both behaviors, and engaged in neither was determined. The mean group size was determined for all sightings as well as for sightings with begging or trawler associations observed.

### Social analyses

Analyses of association were conducted for non-calf individuals that were identified on ≥ 6 field days. Coefficients of association (COA) or association indices are estimates of how much time two individuals are seen together, usually expressed as a proportion. The half-weight index (HWI) of association was used to reduce the bias resulting from the fact that not all individuals in a group are always identified by photo-identification [32–33]. The equation for HWI is:

$$\frac{x}{x+\frac{1}{2}\left(n_{A}+n_{B}\right)}$$

Where

- *x* is the number of sightings animals A and B were seen together
- *n*_A_ is the number of sightings in which only animal A was identified
- *n*_B_ is the number of sightings in which only animal B was identified

A resulting value of 1 means that the animals were seen together in all sightings; whereas, a value of 0 indicates the animals were never sighted together.

One of the assumptions of association indices is that “if one individual is identified in a sample period, then all its associates are identified” [34]. Therefore, in larger study areas where the entire area is not covered during each survey, setting the sampling period to a longer time period is important. The sampling period in this study was variable, with each period based on the number of days it took to cover the entire study area. Therefore, each sampling period was assigned manually, and ranged from 5 to 12 field days. Survey days were not consecutive; therefore, it is possible that animals may have moved within the duration of the sampling period.

Half-weight indices of association were calculated for each pair of individuals (dyad) using SOCPROG 2.4 [35]. To test the accuracy of the calculated association indices versus the true amount of time that individuals spent together, a likelihood approximation of the correlation between the true and estimated association indices was calculated [34]. The mean HWIs for dyads were compared within and between classes for the classes of begging and associating with trawlers. A Mantel test was used to test for significance in the difference in association levels within classes compared to association levels between classes.

An estimate of social differentiation was calculated to determine the amount of homogeneity versus differentiation within the social system based on the coefficient of variation (CV) of the true association indices using the likelihood method [34]. This test indicated whether or not there was social structure within the group of animals analyzed. Further testing then attempted to identify what that structure looked like and how individuals were broken into groups or clusters.

Community division by modularity was used in SOCPROG 2.4 to cluster animals into groups based on association indices. Community division by modularity is based on the difference between the observed proportion of associations in the cluster compared to the expected proportion of associations [36]. If the modularity value was greater than 0.3, the clusters represented a useful division within the group of animals used for analysis [36].

### Utilization areas

The locations of sightings were plotted in ArcGIS 9.3. Utilization areas were created using kernel density analysis using Home Range Tools for ArcGIS Version 1.1 [37]. A utilization area was created for each social cluster. Utilization areas were created with 50% and 90% isopleths, representing the area in which the dolphin would be expected to spend that percentage of its time. An adaptive kernel was used, and the smoothing parameter was set to 0.8 proportion of reference bandwidth.

## Results

Common bottlenose dolphins associated with a shrimp trawler in 13 sightings across 5 days and begged in 131 sightings across 65 days. There were 137 non-calf dolphins seen on ≥ 6 days and used in subsequent analyses. Overall 86 individuals (62.8%) were observed engaging in one or both human-interaction behaviors. Fifty-one (37%) were trawler dolphins, and 86 (63%) were non-trawler dolphins. Fifty-five (40%) of the dolphins were beggars, 71 (52%) were non-beggars, and 11 (8%) were of unknown beg status. Only 20 individuals (15%) engaged in both behaviors. Forty (29%) of the dolphins did not engage in either human-related foraging behavior, and the 11 (8%) with unknown beg status were all non-trawler dolphins.

The mean group size was 5.8 ± 7.6 for all sightings. The mean group size for trawler-associated sightings was 29.5 ± 22.3, and based on our definition of a trawler sighting, all dolphins in the sighting associated with the trawlers. For sightings with begging, the mean group size was 9.3 ± 10.7, and the average number of dolphins that begged was 2.2 ± 2.7. In 57.3% of sightings with begging, only one dolphin was observed begging (75 of 131 beg sightings) despite the overall group size of 1–39 dolphins present. Although sex and age class was unknown for the majority of individuals, we observed males, females, and calves throughout the study, including in sightings with begging and with trawler interactions.

### Social analyses

The estimate of the correlation coefficient between the true association indices and the estimated association indices was 0.435 (S.E. 0.047) using the likelihood approximation. Estimates could range from 0–1, and a value of 0.435 means that the association indices obtained were somewhat representative of the true associations [34]. Therefore, the results of this study were useful for generalizations, but association indices between specific pairs of individuals may not have been valid. The overall mean half-weight index (HWI) was 0.05 ± 0.03, with a mean maximum HWI of 0.53 ± 0.20 (Table 1).

**Table 1:** Summary of half-weight association indices for common bottlenose dolphins *Tursiops truncatus* near Savannah, Georgia.

	Mean	Max		Mean	Max
**NT**	0.03 (0.01)	0.47 (0.18)	**NB**	0.06 (0.03)	0.55 (0.19)
**T**	0.09 (0.03)	0.62 (0.19)	**B**	0.05 (0.03)	0.53 (0.22)
**NT-NT**	0.04 (0.02)	0.44 (0.19)	**NB-NB**	0.07 (0.04)	0.49 (0.19)
**T-T**	0.20 (0.07)	0.60 (0.20)	**B-B**	0.05 (0.03)	0.46 (0.22)
**NT-T**^a^	0.02 (0.03)	0.21 (0.16)	**NB-B**^a^	0.05 (0.03)	0.42 (0.19)
**T-NT**^a^	0.02 (0.01)	0.35 (0.14)	**B-NB**^a^	0.05 (0.04)	0.41 (0.19)
**Within**	0.10 (0.09)	0.50 (0.20)	**Within**	0.06 (0.04)	0.46 (0.21)
**Between**	0.02 (0.03)	0.26 (0.17)	**Between**	0.05 (0.03)	0.42 (0.18)
**Overall**	0.05 (0.03)	0.53 (0.20)	**Overall**	0.05 (0.03)	0.53 (0.20)

The mean and maximum half-weight association indices for non-trawler (NT) and trawler (T) common bottlenose dolphins *Tursiops truncatus* near Savannah, Georgia, as well as non-begging (NB) and begging (B) dolphins. Standard deviations are in parentheses.

^a^There is no biological difference between NT-T and T-NT or NB-B and B-NB; this distinction is an artifact of the calculations.

**Fig 2:**
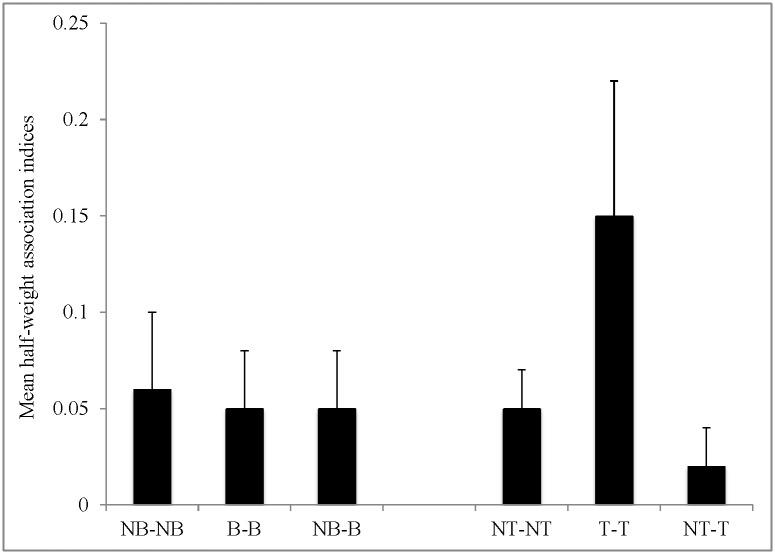
Mean half-weight association indices between pairs of common bottlenose dolphins *Tursiops truncatus* near Savannah, Georgia that associate with trawlers (T) and do not associate with trawlers (NT), and separately for dolphins that are beggars (B) and non-beggars (NB).

The mean HWI was higher for trawler dolphins than non-trawler dolphins (Table 1). The mean HWI was highest between pairs of trawler dolphins (0.20 ± 0.07) compared to pairs of non-trawler dolphins (0.04 ± 0.02) and mixed pairs (0.02 ± 0.02; Fig 2). In addition, maximum HWI was greatest for trawler pairs (0.60 ± 0.20), followed by non-trawler pairs (0.44 ± 0.19) and then mixed pairs (T-NT 0.35 (SD 0.14) and NT-T 0.21 (SD 0.16); Table 1). There was a significant difference in association indices between animals that belonged to the same trawler class compared to animals that belonged to different trawler classes (Mantel test t = 16.3244, p < 0.0001, Matrix correlation 0.25194; Table 1).

There was little difference in mean HWI for non-beggars versus beggars, in contrast to what was observed between non-trawler and trawler dolphins (Table 1). The mean HWI for pairs of non-beggars (0.07 ± 0.04) was slightly greater than the mean HWI for pairs of beggars (0.05 ± 0.03) and mixed pairs (0.05 ± 0.04; Fig 2). Maximum HWI ranged between 0.41–0.55 for all beg classes (Table 1). According to the tests for differences in associations between/within classes, there was a significant difference in association indices between animals that belonged to the same beggar class compared to animals that belonged to different beggar classes (Table 1; Mantel test t = 3.7235, p = 0.0001, Matrix correlation 0.056412).

The bottlenose dolphins near Savannah had a well-differentiated social structure, with a social differentiation value of 0.902 (S.E. 0.067) using the likelihood method. Community division by modularity produced 6 clusters (Table 2) and a modularity of 0.307, which indicated that the grouping was a useful division. Three of the clusters were comprised entirely of non-trawler dolphins (Clusters 1, 2, and 3; Table 1). Two clusters were comprised of almost all trawler dolphins, with two non-trawler dolphins in a cluster of 30 animals and 6 in a cluster of 27 animals (Clusters 4 and 5; Table 1). One cluster only contained 2 dolphins, both of which were trawler animals (Cluster 6; Table 1). Begging was mixed throughout the clusters, with 5 of the 6 clusters containing both beggars and non-beggars. Cluster 6 only contained 2 individuals, both of which were beggars.

**Table 2:** Six social clusters of common bottlenose dolphins *Tursiops truncatus* near Savannah, Georgia.

Cluster	% Trawler	% Beggars	Total
**1**	0%	32%	22
**2**	0%	50%	34
**3**	0%	41%	22
**4**	93%	37%	30
**5**	78%	33%	27
**6**	100%	100%	2

Since individual dolphins can exhibit both begging and trawler association behaviors, or neither behavior, the numbers will not add to 100%.

### Utilization areas

**Fig 3:**
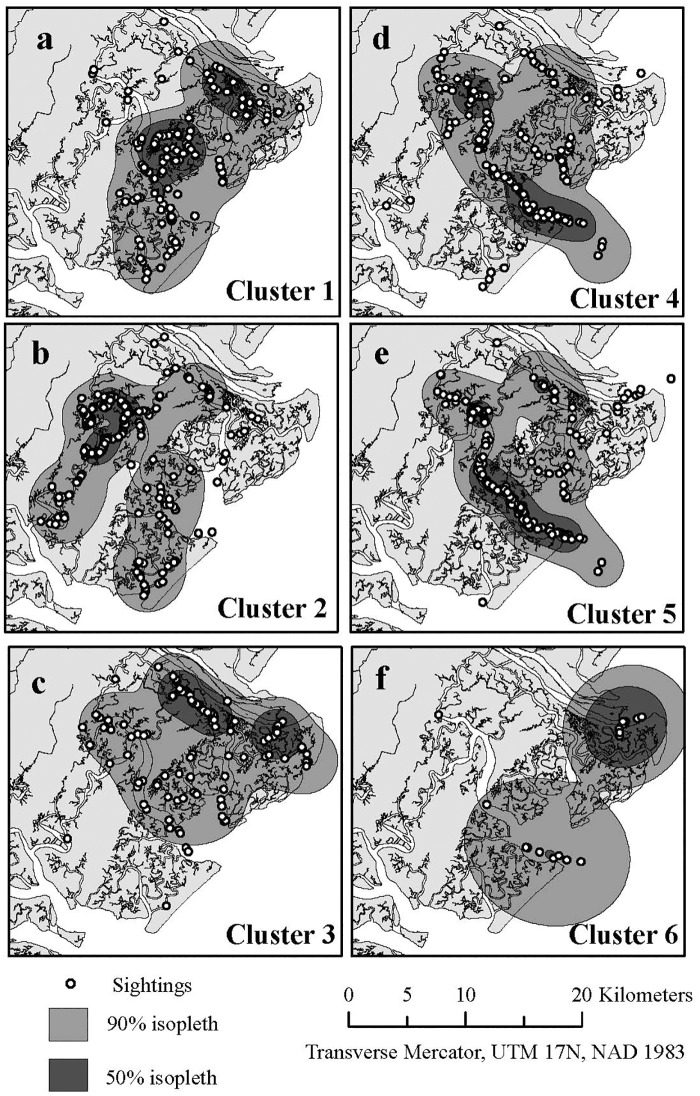
Utilization areas for social clusters of common bottlenose dolphins *Tursiops truncatus* near Savannah, Georgia. Clusters 1, 2, and 3 were considered non-trawler clusters, and clusters 4, 5, and 6 were considered trawler clusters.

Social clusters 1, 2, and 3 were the non-trawler clusters. The utilization area of cluster 1 covered many of the smaller rivers and creeks in the center portion of the study area (Fig 3a). Social cluster 2 was found in many similar sized bodies of water (Fig 3b); however, the utilization area was primarily found in the western portion of the study area. The dolphins in social cluster 3 were found in the northern portion of the study area, with a core utilization area in Bull River and Lazaretto Creek (Fig 3c). Social clusters 4, 5, and 6 were the trawler clusters. Clusters 4 and 5 had similar utilization areas with core usage in the large Wassaw Sound and Wilmington River (Fig 3d and 3e). Cluster 6 was comprised of only 2 dolphins, which were sighted in both Wassaw Sound and Lazaretto Creek but had a core utilization area centered near the mouth of Lazaretto Creek (Fig 3f).

## Discussion

Both trawler associations with and begging behaviors by dolphins were prevalent in the waterways around Savannah. The high mean coefficient of association for pairs of trawler dolphins as well as the splitting of social clusters revealed that the social structure of dolphins near Savannah was differentiated based on associations with trawlers. However, beggars were mixed throughout social clusters, indicating that this behavior was not related to the observed social structure.

The common bottlenose dolphins near Savannah live in a well-differentiated society with an overall mean HWI of 0.05 ± 0.03. Similar mean levels of association have been observed within bottlenose dolphin groups in locations where some of the individuals are known to engage in a human-related foraging behavior [9–10]. Association levels between dolphins near Savannah differed based on whether or not individuals associated with trawlers. There was a significant difference in the within (0.10 ± 0.09) and between (0.02 ± 0.03) class association levels for classes of trawler and non-trawler dolphins, meaning that dolphins spent more time with other dolphins of similar trawler classification (*i*.*e*., trawler or non-trawler) than they did with dolphins dissimilar to them. While there was also a significant difference in the within (0.06 ± 0.04) and between (0.05 ± 0.03) class association levels for beggars and non-beggars, the differences were much smaller than those observed for trawler and non-trawler dolphins. The HWI provided evidence that common bottlenose dolphins near Savannah associated more with dolphins of the same trawler status than they did with dolphins of the same beggar status. This was confirmed by the composition of the six social clusters that were formed as a result of community division through modularity. These six social clusters were split into trawler and non-trawler groups; however, begging dolphins were mixed throughout the clusters.

The question that remains is: why are trawler dolphins grouped together, but beggars are spread throughout the clusters? The division of social groups based on trawler interaction status indicates that trawler foraging may be a socially learned behavior, as the horizontal transmission of specialized foraging behaviors has been recorded in cetaceans [13–15]. However, due to the sporadic nature of beggars throughout the clusters, the mechanisms for the propagation of begging are less clear. Bottlenose dolphins that learned to accept food handouts from boats in south-western Australia showed correlations to both the use of high-density boat areas as well as higher coefficients of associations with previously conditioned dolphins [14]. Social learning may still be a factor in the propagation of both behaviors, but distinctions in the techniques used and caloric outcomes may result in differences in the way the behaviors are spread throughout the social clusters.

The energy expenditure versus the reward may not be equal for the behaviors of begging and associating with trawlers. There is a greater amount of food available in trawler nets compared to the amount that is usually handed out to begging dolphins, if they are fed at all. Dolphins feeding around fish farms near Sardinia, Italy had lower association strengths than dolphins that did not feed near the fish farms, possibly because there would be an increase in competition if more individuals were involved [8]. However, dolphins that engaged in human-related foraging behaviors around aquaculture farms and trawlers near Lampedusa, Italy had the highest association levels of any of the dolphin social clusters in the study [9]. It was hypothesized that by associating with individuals that forage in the same manner, the dolphins expand their efficiency and safety, particularly while interacting with trawler nets [9]. Therefore, having associates that forage in the same manner may be more important for an activity such as trawler foraging, which may involve group manipulation of nets and a higher payout, than it is for begging, which may be a more solitary activity as was observed in 57.3% of beg sightings in the current study.

Group size may also serve as an indication of the significance of cooperative foraging or the size of the reward. Killer whales in the eastern North Pacific are divided into mammal-eating transients and fish-eating residents. The group sizes of these two groups differ, possibly due to the reliance on cooperative hunting as well as the resulting prey size for transients [3]. Although cooperative foraging may or may not be a significant benefit to dolphins feeding from trawlers, the behaviors of begging and associating with trawlers do differ in the amount of prey captured and therefore, the carrying capacity of each behavior. Sightings in which dolphins associated with trawlers near Savannah were triple the size of sightings in which dolphins begged.

Overall, homophily, or behavior matching, may be either neutral or positive for trawler dolphins, whereas it may be negative for beggars. It may be advantageous for individuals to have associates that engage in different foraging behaviors, as they will have less competition within the group and therefore greater availability of prey for all members [3,19]. Feeding around trawlers likely provides a large food source available for a long period of time. However, begging may result in a sporadic and smaller food source, and therefore it may only be beneficial for individuals or small groups.

The social clustering of dolphins based on trawler foraging but not begging may also be partially due to access to and predictability of the food source. Specifically, trawlers are clustered in space and time, whereas recreational boats have less predictable locations and timing. Only the utilization areas of trawler dolphins overlapped with the areas where trawlers operated, in the ocean. Therefore, associating with trawlers for food is only available to dolphins in certain areas, whereas beggars could be found anywhere that either trawlers or recreational boats are present, including the rivers, sounds, and small creeks.

The social clusters of common bottlenose dolphins near Savannah had different but overlapping utilization areas. The non-trawler clusters were found primarily in creeks and smaller, more inland portions of rivers. In contrast, the trawler clusters were found in Wassaw Sound and the lower Wilmington River, which are much larger bodies of water and a transit area for shrimp trawlers. Bottlenose dolphin clusters in other areas worldwide utilized areas with different habitat types or environmental variables, such as water depth, bottom type, and distance to shore [5,38–40]. The trawler group in Moreton Bay, Australia occupied deeper waters that were farther from shore, whereas the non-trawler dolphins were sighted closer to shore in shallower water in areas covered by seagrass [40]. Despite these differences the trawler and non-trawler groups in Moreton Bay still shared 31% and 14.5% of their core utilization areas, respectively [6], similar to the overlap observed in the utilization areas of the trawler and non-trawler clusters in Savannah.

The extent to which the utilization area of dolphins overlapped with that of active trawlers may have played a factor in which groups associate with trawlers, as only the trawler dolphins’ core utilization area at the mouth of Wassaw Sound overlapped with an area of active trawling in this study. Nevertheless, the influence of fisheries on the social structure of dolphin groups should not be disregarded. Extreme social segregation of trawler and non-trawler dolphins in Moreton Bay, Australia was observed in the 1990s [6]. Since then, changes to fisheries rules have led to a decrease in trawling in the area, and the social network of the dolphins has become less differentiated [41]. The trawler and non-trawler dolphins are now spread throughout the social network, suggesting that the trawler associations were a driving force in the earlier differentiation of the social network rather than solely habitat preferences or geographic location in Moreton Bay, Australia [41].

Dolphins in Shark Bay, Australia demonstrated a correspondence between ecological factors and certain foraging tactics, especially the use of sponges as tools, whereas other behaviors showed no correspondence [12]. Foraging tactics may be specific to habitats or prey availability and those habitats, as there is no point engaging in a foraging behavior that will not be successful in a certain type of habitat. However, just because a tool or behavior is available to an individual does not mean that it will necessarily employ it, as only 12% of dolphins sighted in areas where sponges were present were seen carrying sponges [12]. Thereby, ecology alone does not dictate whether certain individuals will engage in a particular behavior.

Based on the differentiation of trawler and non-trawler dolphins into different social clusters, it appears that the behavior of foraging in association with shrimp trawlers is a socially learned behavior. In contrast, begging behavior was observed throughout social clusters, which would indicate a lesser reliance on social learning for the spread of the behavior. However, it is important to consider more factors associated with each behavior. Quantifications of social interactions between individuals are often used in studies examining the potential spread of foraging behaviors through social learning [14–15]. Human-interaction behaviors are often analyzed together; however, parsing different behaviors may alter our understanding of human impacts on social structure, behavior, and foraging success.

## Supporting Information

S1 Fig Discovery curve indicating the number of sightings until each individual was observed begging. Previous research in Savannah indicated that 90% of cataloged beggars displayed begging behavior by their 4th sighting [31]. These data were used to determine that 6 sightings on separate days were sufficient to determine if an individual was a beggar. Therefore, each individual in the social analyses was observed in a minimum of 6 sightings.
(TIF)

S1 Dataset Data used in all analyses. All sighting data including sighting locations and human-interaction behaviors observed and dolphin group size; sighting data by individual dolphins; beg and trawler status for each dolphin; and social clusters with beg and trawler status.
(XLSX)

## References

[1] Schoener TW. Theory of feeding strategies. Annu Rev Ecol Syst. 1971;2: 369–404.

[2] Futuyma DJ, Moreno G. The evolution of ecological specialization. Annu Rev Ecol Syst. 1998;19: 207–233.

[3] Baird RM, Abrams PA, Dill LM. Possible indirect interactions between transient and resident killer whales: implications for the evolution of foraging specializations in the genus *Orcinus*. Oecologia. 1992;89125–132. 10.1007/BF00319024 28313404

[4] Bolnick DI, Svanbäck R, Fordyce JA, Yang LH, Davis JM, Hulsey CD, et al The ecology of individuals: incidence and implications of individual specialization. Am Nat. 2003;161:1–28. 10.1086/343878 12650459

[5] Torres LG, Read AJ. Where to catch a fish? The influence of foraging tactics on the ecology of bottlenose dolphins (*Tursiops truncatus*) in Florida Bay, Florida. Mar Mamm Sci. 2009;25: 797–815.

[6] Chilvers BL, Corkeron PJ. Trawling and bottlenose dolphins' social structure. Proc R Soc Lond B Biol Sci 2001;268: 1901–1905. 10.1098/rspb.2001.1732 PMC1088825 11564345

[7] Fleming KH. The social structure, behavior, and occurrence of bottlenose dolphins in relation to shrimp trawlers in Southport, North Carolina. M.Sc. Thesis, University of North Carolina at Wilmington. 2004.

[8] Díaz López B, Bernal Shirai JA. Marine aquaculture and bottlenose dolphins’ (*Tursiops truncatus*) social structure. Behav Ecol Sociobiol. 2008;62: 887–894.

[9] Pace DS, Pulcini M, Triossi F. Anthropogenic food patches and association patterns of *Tursiops truncatus* at Lampedusa Island, Italy. Behav Ecol. 2011;22: 276–298.

[10] Daura-Jorge FG, Cantor M, Ingram SN, Lusseau D, Simões-Lopes PC. The structure of a bottlenose dolphin society is coupled to a unique foraging cooperation with artisanal fishermen. Biol Lett. 2012;8: 702–705. 10.1098/rsbl.2012.0174 22552635 PMC3440962

[11] Nowacek DP. Sequential foraging behavior of bottlenose dolphins, *Tursiops truncatus*, in Sarasota Bay, FL. Behaviour. 2002;139: 1125–1145.

[12] Sargeant BL, Wirsing AJ, Heithaus MR, Mann J. Can environmental heterogeneity explain individual foraging variation in wild bottlenose dolphins (*Tursiops* sp.)? Behav Ecol Sociobiol. 2007;61: 679–688.

[13] Mann J, Stanton MA, Patterson EM, Bienenstock EJ, Singh LO. Social networks reveal cultural behavior in tool-using dolphins. Nat Commun. 2012;3: 980 10.1038/ncomms1983 22864573

[14] Donaldson R, Finn H, Bejder J, Lusseau D, Calver M. The social side of human-wildlife interaction: wildlife can learn harmful behaviors from each other. Anim Conserv. 2012;

[15] Allen J, Weinrich M, Hoppitt W, Rendell L. Network-based diffusion analysis reveals cultural transmission of lobtail feeding in humpback whales. Science. 2013;340: 485–488. 10.1126/science.1231976 23620054

[16] Page RA, Ryan MJ. Social transmission of novel foraging behavior in bats: Frog calls and their referents. Curr Biol. 2006;16: 1201–1205. 10.1016/j.cub.2006.04.038 16782010

[17] Jaeggi AV, Dunkey LP, Van Noordwijk MA, Wich SA, Sura AAL, Can Schaik CP. Social learning of diet and foraging skills by wild immature Bornean Orangutans: Implications for culture. Am J Primatol. 2010;72: 62–71. 10.1002/ajp.20752 19790189

[18] Kendal RL, Custance DM, Kendal JR, Vale G, Stoinski TS, Rakotomalala NT, et al Evidence for social learning in wild lemurs (*Lemur catta*). Learn Behav. 2010;38: 220–234 10.3758/LB.38.3.220 20628161

[19] Giraldeau LA. Group Foraging: the skill pool effect and frequency-dependent learning. Am Nat. 1984;124: 72–79.

[20] Samuels A, Bejder L. Chronic interaction between humans and free-ranging bottlenose dolphins near Panama City Beach, Florida, USA. J Cetacean Res Manag. 2004;6: 69–77.

[21] Finn H, Donaldson R, Calver M. Feeding Flipper: a case study of a human-dolphin interaction. Pac Conserv Biol. 2008;14: 215–225

[22] Perrtree RM, Kovacs CK, Cox TM. Standardization and application of metrics to quantify human-interaction behaviors by the bottlenose dolphin (*Tursiops* spp.). Mar Mamm Sci. 2014;30: 1320–1334.

[23] Powell JR, Wells RS. Recreational fishing depredation and associated behaviors involving common bottlenose dolphins (*Tursiops truncatus*) in Sarasota Bay, Florida. Mar Mamm Sci. 2011:27:111–129.

[24] Kovacs CJ, Cox TM. Quantifications of interactions between common bottlenose dolphins (*Tursiops truncatus*) and a commercial shrimp trawler near Savannah, Georgia. Aquat Mamm. 2014;40: 81–94

[25] Fertl D, Leatherwood S. Cetacean interaction with trawls: A preliminary review. J Northw Atl Fish Sci. 1997;22: 219–248.

[26] Broadhurst MK. Bottlenose dolphins, *Tursiops truncatus*, removing by-catch from prawn-trawl codends during fishing in New South Whales, Australia. Mar Fish Rev. 1998;60: 9–14.

[27] Svane I. Occurrence of dolphins and seabirds and their consumption of by-catch during prawn trawling in Spencer Gulf, South Australia. Fish Res. 2005;76: 317–327.

[28] Gonzalvo J, Valls M, Cardona L, Aguilar A. Factors determining the interaction between common bottlenose dolphins and bottom trawlers off the Balearic Archipelago (western Mediterranean Sea). J Exp Mar Biol Ecol. 2008;367: 47–52.

[29] Shane SH. Behavior and ecology of the bottlenose dolphin (*Tursiops truncatus*) at Sanibel Island, Florida In: Leatherwood S, Reeves RR, editors. The bottlenose dolphin. Academic Press, San Diego; 1990 pp 245–265.

[30] Urian KW, Hohn AAA, Hansen LJ. Status of the photo-identification catalog of coastal bottlenose dolphins of the western North Atlantic: Report of a workshop of catalog contributors. NOAA Tech Memo. NMFS-SEFSC-425; 1999.

[31] Perrtree RM. Begging behavior by the common bottlenose dolphin Tursiops truncatus near Savannah, Georgia: Prevalence, spatial distribution, and social structure. M.Sc. Thesis, Savannah State University, Savannah, GA. 2011.

[32] Cairns SJ, Schwager SJ. A comparison of association indices. Anim Behav. 1987;35: 1454–1469

[33] Smolker RA, Richards AL, Connor RC, Pepper JW. Sex differences in patterns of association among Indian Ocean bottlenose dolphins. Behaviour. 1992;123: 38–69.

[34] Whitehead H. Analyzing Animal Societies: Quantitative methods for vertebrate social analysis. University of Chicago Press, Chicago; 2008.

[35] Whitehead H. SOCPROG programs: analyzing animal social structures. Behav Ecol Sociobiol. 2009;63: 765–778.

[36] Newman MEJ. Analysis of weighted networks. Phys Rev E. 2004;70: 056131. 10.1103/PhysRevE.70.056131 15600716

[37] Rodgers AR, Carr AP, Beyer HL, Smith L, Kie JG. HRT: Home Range Tools for ArcGIS Version 1.1. 2007. Ontario Ministry of Natural Resources, Centre for Northern Forest Ecosystem Research, Thunder Bay, Ontario, Canada

[38] Rossbach KA, Herzing DL. Inshore and offshore bottlenose dolphins (*Tursiops truncatus*) communities distinguished by association patterns near Grand Bahama Island, Bahamas. Can J Zool. 1999;77: 581–592.

[39] Gubbins CM. Association patterns of resident bottlenose dolphins (*Tursiops truncatus*) in a South Carolina estuary. Aquat Mamm. 2002; 28:24–31

[40] Chilvers BL, Corkeron PJ, and Puotinen ML. Influence of trawling on the behavior and spatial distribution of Indo-Pacific bottlenose dolphins (*Tursiops aduncus*) in Moreton Bay, Australia. Can J of Zool. 2013;81: 1947–1955.

[41] Ansmann IC, Parra GJ, Chilvers BL, Lanyon JM. Dolphins restructure social system after reduction of commercial fisheries. Anim Behav. 2012;84: 575–581.

