# Supplementary figures and images for: Social Differentiation in Common Bottlenose Dolphins (Tursiops truncatus) that Engage in Human-Related Foraging Behaviors
Source: PLoS One. 2017 Feb 1;12(2):e0170151. doi: 10.1371/journal.pone.0170151 (PMC5287471; doi:10.1371/journal.pone.0170151)

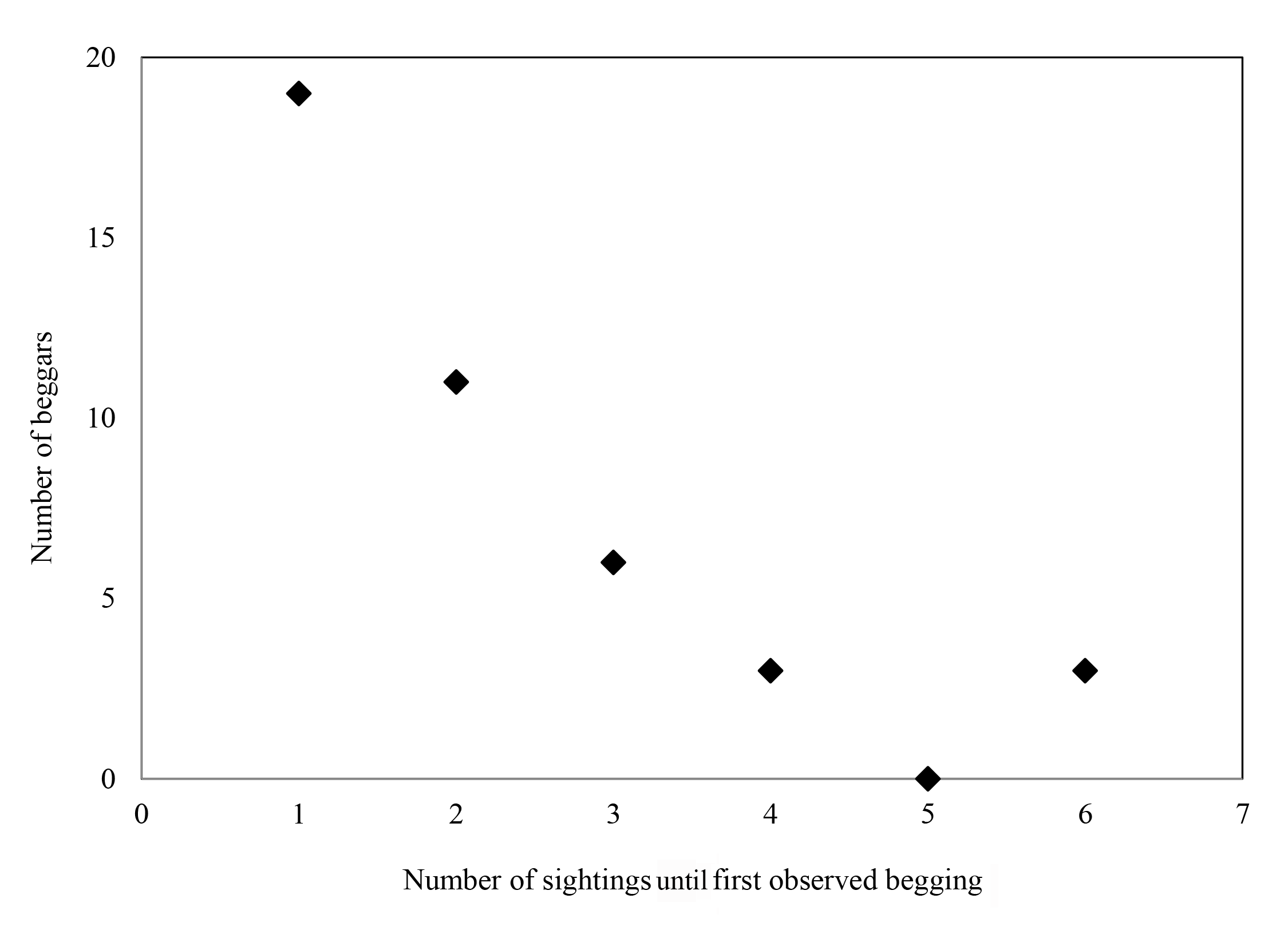

Supplement: S1 Fig — Previous research in Savannah indicated that 90% of cataloged beggars displayed begging behavior by their 4th sighting [31]. These data were used to determine that 6 sightings on separate days were sufficient to determine if an individual was a beggar. Therefore, each individual in the social analyses was observed in a minimum of 6 sightings. (TIF) [file pone.0170151.s001.tif]
